# A semi-automatization magnetic solid-phase extraction method of LC-MS/MS for the quantification of homocysteine and its related metabolites in serum

**DOI:** 10.1016/j.plabm.2026.e00535

**Published:** 2026-05-05

**Authors:** Zhicheng Ye, Xueding Han, Guanfeng Lin, Jianwei Zhou, Yingsong Wu

**Affiliations:** a Institute of Antibody Engineering, School of Laboratory Medicine and Biotechnology, Southern Medical University, Guangzhou, 510515, China; b Guangzhou Darui Biotechnology Co., Ltd., Guangzhou, 510705, China

**Keywords:** Magnetic solid-phase extraction, Semi-automatization, LC-MS/MS, Homocysteine metabolism cycle, Diagnosis

## Abstract

**Background:**

Clinical mass spectrometry is recognized for its high specificity and sensitivity in quantifying small-molecule biomarkers in serum. However, its broad adoption in clinical settings has been limited by challenges such as low automation and time-consuming workflows.

**Methods:**

This study aimed to develop a rapid, sensitive, and automated magnetic solid-phase extraction(MSPE) method using Hydrophilic-Lipophilic Balance (HLB) magnetic bead-based sample preparation coupled with liquid chromatography–tandem mass spectrometry (LC–MS/MS) for the simultaneous quantification of homocysteine(Hcy) and its related nine metabolites. The method was comprehensively validated for specificity, linearity, sensitivity, accuracy, precision, matrix effects, carry-over; and compared it with solid-phase extraction (SPE) methods.

**Results:**

Results showed excellent linearity (r^2^ > 0.995) for all nine biomarkers associated with the homocysteine metabolic cycle. Both the limits of detection and quantification met the clinical requirements. Recoveries at low, medium, and high spiking levels ranged from 85.46% to 114.48%. Intra-day precision (CV) was between 0.82% and 7.63%, and inter-day precision (CV) ranged from 1.62% to 11.43%. Matrix effects were acceptable, with internal standard–normalized matrix factors ranging from 0.83 to 1.19. Carry-over rates were between −7.43% and 5.21%. Method comparison with protein precipitation sample preparation showed correlation coefficients from 0.9462 to 0.9957, indicating no systematic bias.

**Conclusions:**

In conclusion, the developed semi-automatization method is rapid, highly sensitive, and reproducible, making it suitable for quantitative analysis of these nine homocysteine cycle–related biomarkers in clinical serum samples. It provides a reliable analytical tool for the diagnosis, treatment, and prevention of associated diseases.

## Highlights

• This method enables semi-automatization with cost-effectiveness, greatly facilitating its clinical implementation.
• A high-throughput LC-MS/MS quantifies Hcy and nine metabolic biomarkers in a single run, which facilitates the systematic monitoring and diagnosis of diseases.
• Comparison with the LC-MS/MS method employing protein precipitation pretreatment demonstrates strong correlation with minimal differences.

## 1 Introduction

Liquid chromatography-tandem mass spectrometry (LC-MS/MS) exhibits exceptional specificity and sensitivity, commonly used in microanalysis and trace analysis [1]. It is extensively applied across various fields, including clinical diagnostics, food safety, environmental monitoring, and forensic toxicology [1]^,^ [2]. In clinical diagnostics, LC–MS/MS plays a critical role in biomarker quantification, newborn screening for inherited metabolic disorders (IMDs), and therapeutic drug monitoring (TDM) [3]^,^ [4]. Although numerous LC–MS/MS methods have been developed for clinical biomarker detection [5], existing sample pretreatment approaches—such as protein precipitation (PPT), liquid-liquid extraction (LLE), and conventional solid-phase extraction (SPE)—remain labor-intensive, time-consuming, and challenging to automate [6]^,^ [7].

**Fig. 1:**
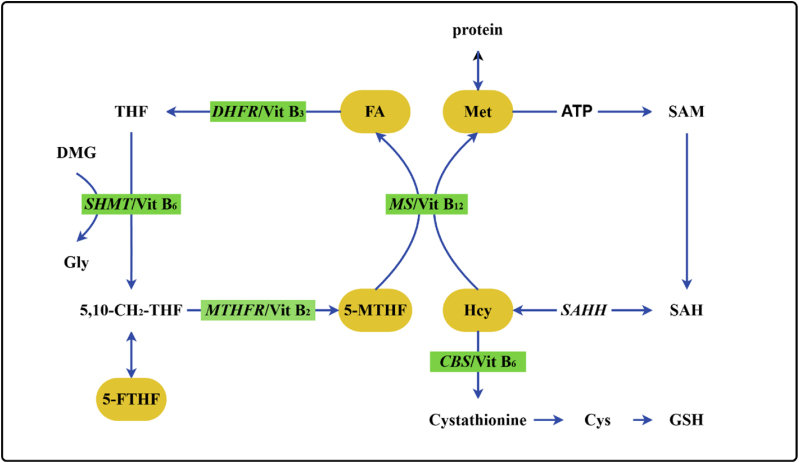
Schematic diagram of remethylation and transsulfuration pathways.

Folate and its active metabolites (5-MTHF, 5-FTHF) constitute the core carriers for intracellular one-carbon moiety transfer [8]. They participate directly in nucleotide synthesis, DNA methylation, and amino acid metabolism through the folate cycle [9]. Hcy is a sulfur-containing amino acid, exclusively generated as a demethylation product of methionine(Met) [10]. Hcy and its metabolites represent pivotal nodes in one-carbon metabolism and the methylation cycle [11]. Hcy links the folate cycle, methionine cycle, and antioxidant systems through its two major metabolic pathways: remethylation and transsulfuration. These interconnected metabolic pathways depend on the coordinated regulation of vitamins B_2_ (riboflavin), B_3_ (niacin), B_6_ (pyridoxine), and B_12_ (cobalamin) [12]. Metabolic imbalance can lead to Hcy accumulation (>15 μmol/L) [13], which impairs vascular endothelial function and increases the risk of stroke and cardiovascular diseases [14]; Concurrently, insufficient synthesis of S-adenosylmethionine (SAM) results in aberrant DNA methylation, which is closely associated with neurodegenerative disorders (e.g., Alzheimer's disease), fetal developmental abnormalities, and carcinogenesis [15]^,^ [16]. (Fig. 1).

Integrated quantification of folate and homocysteine metabolites is essential for auxiliary diagnosis of hyperhomocysteinemia and cardiovascular risk stratification [14]. While immunoassays are amenable to convenient, they are susceptible to cross-reactivity among metabolites, exhibit lower sensitivity, and lack multiplexing capability for simultaneous multi-analyte detection [17]. Conventional LC-MS/MS methods offer high sensitivity and superior anti-interference capacity [18]. However, their sample pretreatment relies on PPT, which required manual operation by laboratory personnel [19]^,^ [20].

To achieve low-cost semi-automatization and high-throughput detection of the following analytes: folate, 5-MTHF, 5-FTHF, Hcy, methionine (Met), vitamin B_2_, vitamin B_3_, vitamin B_6_, and vitamin B_12_, this study develop a novel methods: Automated serum sample pretreatment using modified HLB magnetic beads processed via an automated nucleic acid extraction instrument. This study pioneers the automated quantification of 9 biomarkers spanning the folate and homocysteine metabolic pathways, thereby providing a laboratory foundation for precision medicine.

## 2 Experimental

### 2.1 Materials and chemicals

The standards of folate, 5-MTHF, 5-FTHF, Hcy, methionine (Met), vitamin B_2_, vitamin B_3_, vitamin B_6_, and vitamin B_12_ were purchased from Sigma-Aldrich (St. Louis, MO, USA) Trading Co., Ltd. Isotope labeled internal standards of folate-^13^C_5_, 5-MTHF-^13^C_5_, 5-FTHF-^13^C_5_, Hcy-d_4_, Met-d_3_, vitamin B_2_-^15^N_2_,^13^C_4_, vitamin B_3_-^15^N,^13^C_3_ and vitamin B_6_-d_3_ were purchased from Cambridge Isotope Laboratories (Tewksbury, MA, USA). Citric acid monohydrate and sodium citrate dihydrate were purchased from Sigma-Aldrich (St. Louis, MO, USA). Bovine serum albumin (BSA) was purchased from Sigma-Aldrich (St. Louis, MO, USA). Ascorbic acid was purchased from Sigma-Aldrich (St. Louis, MO, USA). Dithiothreitol(DTT) was purchased from Merck (Darmstadt, Germany). 5-Sulfosalicylic acid was purchased from Merck (Darmstadt, Germany). Reconstituted solution(0.25% potassium cyanide 1M sodium hydroxide) were purchased from Guangzhou Darui Biotechnology Co., Ltd (Guangzhou, Guangdong Province, China). Formic acid was purchased from Merck (Darmstadt, Germany). HLB magnetic bead was purchased from beaver (Suzhou, Jiangsu Province, China). Methanol was purchased from Anpel (Shanghai, China), ethanol was purchased from Sigma-Aldrich (St. Louis, MO, USA), acetonitrile was purchased Thermo Fisher (St. Louis, MO, USA), ultrapure water is obtained by purified water equipment. In vitro diagnostic kit for complete folic acid and homocysteine and its metabolites (protein precipitation method) was purchased from Guangzhou Darui Biotechnology Co., Ltd (Guangzhou, Guangdong Province, China).

### 2.2 Preparation of standard solutions

The stock solutions of folate, 5-MTHF, 5-FTHF, vitamin B_2_, vitamin B_3_, vitamin B_6_, and vitamin B_12_ were prepared separately at 1 mg/mL in 50% methanol; the stock solution of Hcy was prepared at 10 mg/mL in 5% HCl and the stock solution of Met was prepared at 10 mg/mL in purified water, the corresponding isotope labeled internal standards were handled in the same manner, they were stored at −20 °C. 5% BSA solution was prepared by dissolving 50 g BSA, 13.77 g citric acid monohydrate, 10.15 g sodium citrate dihydrate and 1 mL ascorbic acid in 900 mL deionized water, and then the volume was adjusted to 1000 mL. The standard solutions were serially diluted with 5% BSA solution to generate the calibration curves. The calibration ranges were 2.2 to 120 μmol/L for Hcy; 3.3 to 180 μmol/L for Met; 0.8 to 42.9 nmol/L for folate; 0.74 to 39.85 nmol/L for 5-FTHF; 1.52 to 82.12 nmol/L for 5-MTHF; 3.2 to 171.8 nmol/L for vitamin B_2_; 16.2 to 1317 nmol/L for vitamin B_3_; 3.8 to 205.4 nmol/L for vitamin B_6_; 0.22 to 11.9 nmol/L for vitamin B_12_. The internal standards were prepared in 5% BSA at 20 nmol/L for folate-^13^C_5_, 5 nmol/L for 5-MTHF-^13^C_5_, 2 nmol/L 5-FTHF-^13^C_5_, 10 μmol/L for Hcy-d_4_, 10 μmol/L for Met-d_3_, 50 nmol/L for vitamin B_2_-^15^N_2,_^13^C_4_, 100 nmol/L for vitamin B_3_-^15^N,^13^C_3_ and 10 nmol/L for vitamin B_6_-d_3_. 1 g HLB magnetic beads was dissolved in 10 mL of 25% ethanol, the concentration is 100 mg/mL. Dissociative agent contains 0.5 M citric acid buffer solution and 200 mM DTT. 2% 5-Sulfosalicylic acid was prepared by dissolving 2 g of 5-sulfosalicylic acid in 98 mL of ultrapure water. 25 mL of acetonitrile and 75 mL of ultrapure water prepared as 25% acetonitrile.

### 2.3 Optimization of the amount of magnetic beads

To achieve optimal enrichment efficiency, the optimal amount of magnetic beads was investigated. Specifically, 1 mg, 2 mg, 3 mg, 4 mg, 5 mg, 6 mg, 7 mg, and 8 mg of magnetic beads were used to enrich high concentrations of the standards, and the signal intensities of the standards under different bead amounts were compared. The optimal amount of magnetic beads was selected to maximize the signal intensity for each analyte.

### 2.4 Optimization of the eluting solvent

The elution efficiency of different acetonitrile concentrations for the standards was also investigated to determine the optimal elution concentration. Elution was performed using 5%, 10%, 15%, 20%, 25%, 30%, 35%, and 40% acetonitrile after enrichment, and the analyte signals under different elution concentrations were compared. The optimal acetonitrile concentration was selected to maximize the signal intensity for each analyte.

### 2.5 Pretreatment method

The pretreatment procedure was performed using a 96-well deep-well plate (96 × 2.2 mL). 200 μL of standard, quality control, or serum was added to column 3 or 9 of the 96-well plate. Then, 80 μL of internal standard (diluted ten-fold with dissociative agent) was added to the same wells. After standing for 10 min, 100 μL of reconstituted solution and 500 μL of 2% 5-sulfosalicylic acid were added.

For automated extraction, the following reagents were dispensed into the specified columns of the same plate: 50 μL of HLB magnetic beads (100 mg/mL) and 300 μL of methanol into columns 1 and 7; 300 μL of ultrapure water into columns 2, 4, 8 and 10; 200 μL of ultrapure water into columns 5 and 11; and 100 μL of 25% acetonitrile into columns 6 and 12.

**Fig. 2:**
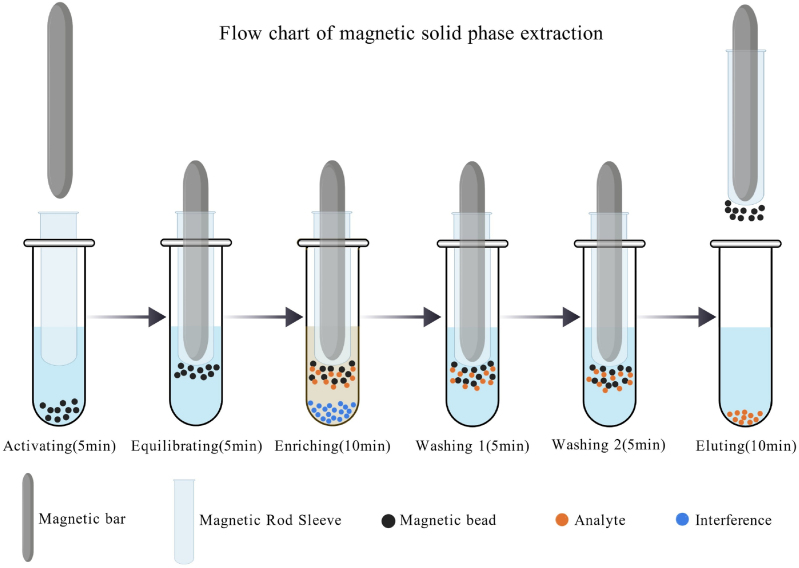
Flowchart of magnetic solid-phase extraction.

The plate was then placed on a multi-tube vortexer and shaken at 1500 rpm for 25 min. Subsequently, the deep-well plate together with an 8-channel magnetic rod sleeve was loaded into a nucleic acid extractor (Stream SP96, Da'an Gene Co., Ltd.). The extraction program was set as follows: magnetic bead activation for 10 min, enrichment for 10 min, washing for 10 min, and elution for 10 min. After extraction, the solution from column 6 was transferred to a 96-well conical-bottom plate (non-coated) (Fig. 2) [21]. The detailed automated extraction procedure for the Stream SP96 is provided in Table S1 of the supplementary material.

### 2.6 LC-MS/MS analysis

Sample analysis was performed using AB SCIEX Triple Quad 4500MD (chromatographic instrument is SCIEX Jasper™ HPLC System) (Framingham, MA, USA). Liquid chromatography separation was conducted with a Waters C18 column (4.6∗50 mm, 2.5 μm) (Milford, MA, USA), column temperature 40 °C. Metabolites and vitamins were separated using a gradient elution program. The mobile phase A was 0.1% formic acid dissolved in water and mobile phase B was methanol. The flow rate was set at 0.8 mL/min. The elution program was set as follows: 0-3 min, 1%-50%B; 3-3.2 min, 50%-98%B; 3.2-4.2 min, 98%B; 4.2-4.5 min, 98%-1%B; 4.5-6 min, 1%B. The automatic sampler was kept at 10 °C. The mass spectrometry conditions for the analyte are multiple reaction monitoring (MRM) with positive electrospray ionization (ESI). The MRM transitions for the target analytes were set as follows: folate: 442.2 → 295.2, 442.2 → 85.2; 5-MTHF: 460.3 → 313.3, 460.3 → 119.4; 5-FTHF: 474.3 → 327.2, 474.3 → 345.5; Hcy: 136.1 → 90.1, 136.1 → 61.1; Met: 150.1 → 104.1, 150.1 → 56.7; vitamin B_2_: 377.2 → 243.3, 377.2 → 216.1; vitamin B_3_: 123.0 → 80.1, 123.0 → 53.1; vitamin B_6_: 184.0 → 148.1, 184.0 → 94.1 and vitamin B_12_: 678.2 → 147.1, 678.2 → 359.1; folate-^13^C_5_: 447.2 → 295.2, 5-MTHF-^13^C_5_: 464.3 → 317.3, 5-FTHF-^13^C_5_: 478.3 → 331.2, Hcy-d4: 140.1 → 94.1, Met-d: 153.1 → 107.1, vitamin B_2_-^15^N_2_,^13^C_4_: 383.2 → 249.3, vitamin B_3_-^15^N,^13^C_3_: 127.1 → 83.1 and vitamin B_6_-d3: 187.0 → 150.1. The following are the main ion source parameters: Curtain Gas (CUR): 30 psi, Collision Gas (CAD): 8 psi, Ion Spray Voltage (IS): 5500 V, Temperature (TEM): 550 °C, Ion Source Gas1 (GS1):65 psi, Ion Source Gas2 (GS2): 65 psi. Detail parameters for HPLC and MRM transitions were given in Table S2 and S3. Instrument control, data collection and data were processed using Analyst MD Software.

### 2.7 Method validation

The performance of the method was validated according to standard guidelines, including linearity, sensitivity, accuracy, precision, matrix effect and carryover. Performance validation is based on the FDA Bioanalytical Method Validation Guidelines [22].

#### 2.7.1 Linearity and sensitivity assessment

Linearity was evaluated by linear regression of peak area ratio (ratio of standard peak area to internal standard peak area) and calibrator concentrations. It was accepted if the correlation coefficient was ≥0.99 and deviation was ≤15%. Limit of detection (LOD) and limit of quantification (LOQ) were calculated to evaluate sensitivity. LOD was defined as the signal-to-noise ratio (S/N) ≥ 3 and coefficient of variation (CV) ≤ 20% for six repeat injections. LOQ was defined as S/N ≥ 10 and CV ≤ 20% for six repeat injections.

#### 2.7.2 Accuracy and precision

Accuracy was evaluated by recovery experiment, low, medium and high concentrations of the standards were spiked in pooled serum from ten healthy individuals. It was accepted that recoveries in the range of 85%–115% and the coefficient of variation ≤15%. Precision was evaluated by analysis of the pooled serum spiked with low, medium and high concentrations of the target standards. Each concentration was measured in six parallel samples on three different days, and the coefficient of variation was required to be ≤ 15%.

#### 2.7.3 Matrix effect and carryover

Matrix effect was evaluated by analysis of 50% methanol solution and serum matrix spiked with low, medium, and high concentrations of standards. The deviation of relative response in the two matrices (methanol and serum) was ≤15%. Carryover was evaluated by alternate injection of low and high concentration samples, The coefficient of variation of the measured low concentrations should be ≤ 15%.

#### 2.7.4 Comparative study

To explore the correlation between magnetic solid-phase extraction (MSPE) and traditional protein precipitation(PPT), clinical samples from Darui Medical Laboratories were tested using both methods. Inclusion criteria were as follows: healthy individuals undergoing routine physical examinations; exclusion of diseases that may significantly affect metabolism, such as severe hepatic or renal dysfunction, thyroid disorders, or malignant tumors; exclusion of patients who had recently used medications that could influence the levels of the analytes, such as folic acid, vitamin B12, vitamin B6, or methotrexate; exclusion of pregnant or lactating women. The sample consisted of 200 cases, with 89 male and 111 female subjects. Clinical samples were serum, stored at −20 °C.

**Fig. 3:**
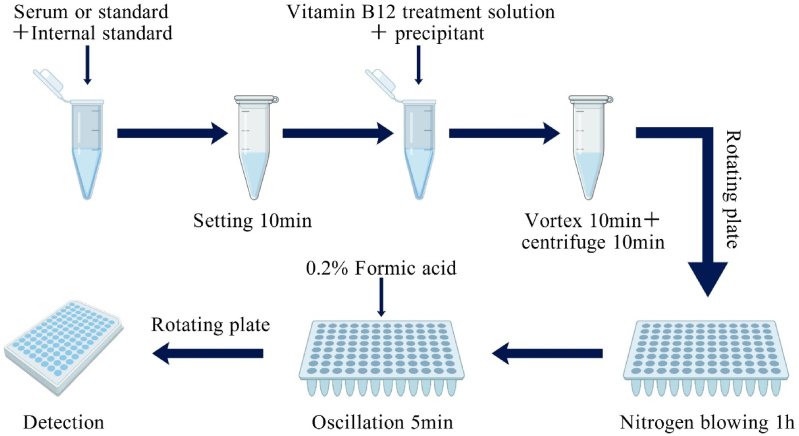
Flowchart of protein precipitation method.

The steps of traditional protein precipitation method are as follows: (1) All samples and reagents were equilibrated to room temperature (10-30 °C) and vortex-mixed thoroughly prior to use. (2) Add 1 mL of purified water to the folate and metabolite internal standard buffer, and vortex until the lyophilized powder is completely dissolved. (3) Mix the reconstituted folate and metabolite internal standard buffer with the folate and metabolite dissociating agent (DTT) at a volume ratio of 1:5 (1.0 mL of folate and metabolite internal standard buffer + 5.0 mL of folate and metabolite dissociating agent) and vortex thoroughly. (4) Pipette 200 μL of serum sample into a 2.0 mL centrifuge tube, add 50 μL of the mixed solution from step (3), vortex-mix, and incubate at room temperature for 10 min. Subsequently, add 100 μL of vitamin B12 treatment solution and 600 μL of folate and metabolite sample precipitant (acetonitrile: methanol = 9:1, V/V). (5) Vortex-mix for 10 min, then centrifuge at 13,000 rpm for 10 min(6) Transfer 300 μL of the supernatant to a 96-well deep-well plate, and dry the sample under nitrogen gas using a nitrogen evaporator (approximately 1 h to complete drying). (7) After drying, add 100 μL of folate and metabolite sample reconstitution solution to the 96-well deep-well plate, seal the plate with an adhesive microplate seal, and shake on a plate shaker for 5 min (8) Remove the adhesive microplate seal, transfer 75 μL of the supernatant to a 96-well conical plate, and seal tightly with an aluminum foil microplate seal. The sample is now ready for liquid chromatography-tandem mass spectrometry (LC-MS/MS) analysis (Fig. 3) [21].

### 2.8 Statistics

Statistic analysis was conducted with Microsoft Excel and method comparison was performed with Passing-Bablok regression and Bland- Altman plots. A p < 0.05 was considered statistically significant.

## 3 Results and discussion

### 3.1 Chromatograms of standard and serum

**Fig. 4:**
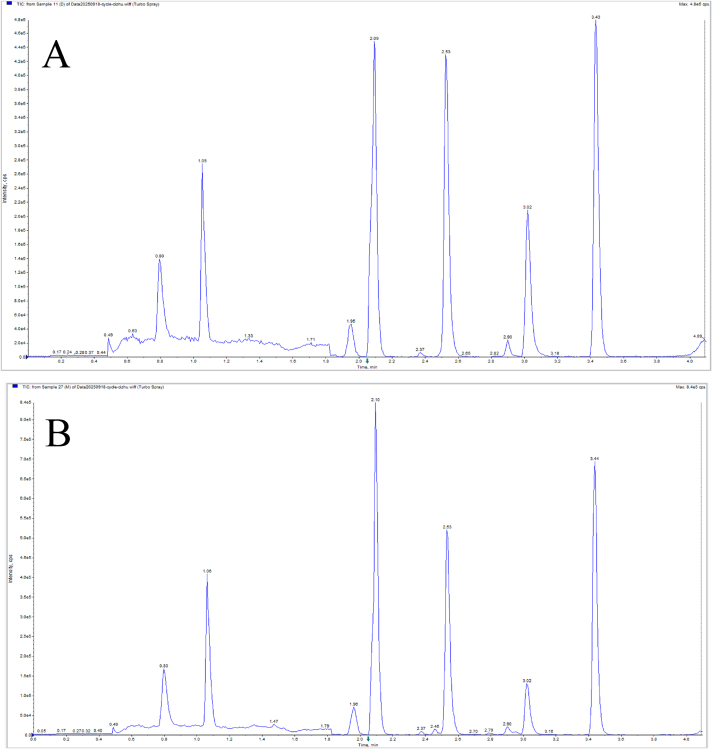
TIC (total ion chromatogram) of standard(A) and serum(B), retention times: 0.80 min for Hcy, 1.07 min for Met, 1.08 min for vitamin B_3_, 1.96 min for vitamin B_6_, 2.09 min for 5-MTHF, 2.53 min for 5-FTHF, 2.90 min for vitamin B_12_, 3.02 min for folate, and 3.43 min for vitamin B_2_.

The optimized magnetic solid-phase extraction (MSPE) method effectively extracted the target analytes from serum. A representative total ion chromatogram shows good separation of all analytes, with the following retention times: Hcy (0.80 min), Met (1.07 min), vitamin B_3_ (1.08 min), vitamin B_6_ (1.96 min), 5-MTHF (2.09 min), 5-FTHF (2.53 min), vitamin B_12_ (2.90 min), folic acid (3.02 min), and vitamin B_2_ (3.43 min) (Fig. 4).

### 3.2 Optimization of concentration of magnetic beads and acetonitrile

**Fig. 5:**
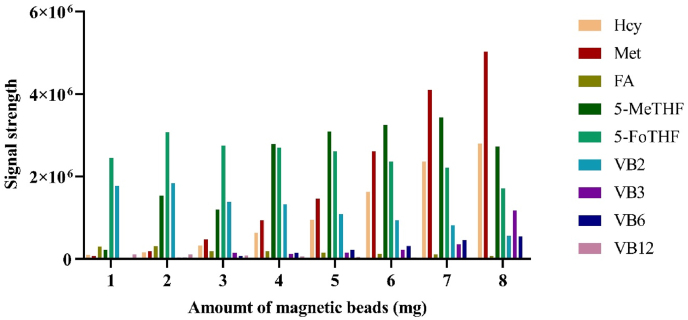
Enrichment effect of different amount of magnetic beads on analytes.

**Fig. 6:**
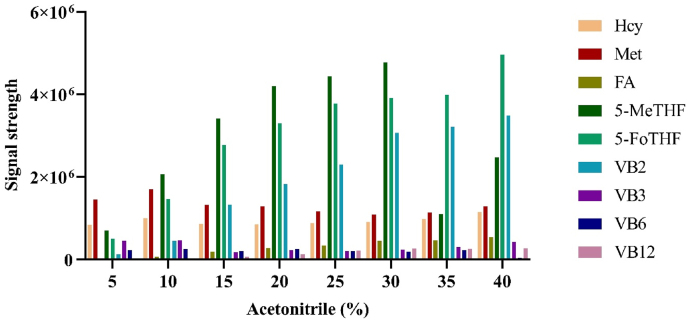
Elution effect of acetonitrile at different concentration on analytes.

Based on a comparison of analyte signal intensities after enrichment with different HLB magnetic bead amounts, 5 mg was determined to be the optimal quantity for maximizing the signal of each analyte (Fig. 5). Similarly, assessment of elution efficiency across a range of acetonitrile concentrations identified 25% as the optimal concentration for producing the highest analyte signals (Fig. 6).

### 3.3 Performance verification

The R^2^ values of linear regression were all greater than 0.995. The LOD of the magnetic SPE method was 0.25 μmol/L for Hcy, 0.2 μmol/L for Met, 0.17 nmol/L for folate, 0.043 nmol/L for 5-MTHF, 0.042 nmol/L for 5-FTHF, 0.08 nmol/L for vitamin B_2_, 12.2 nmol/L for vitamin B_3_, 1.9 nmol/L for vitamin B_6_, 0.074 nmol/L for vitamin B_12_. The LOQ of the magnetic SPE method was 0.5 μmol/L for Hcy, 0.7 μmol/L for Met, 0.34 nmol/L for folate, 0.1 nmol/L for 5-MTHF, 0.25 nmol/L for 5-FTHF, 0.5 nmol/L for vitamin B_2_, 36.5 nmol/L for vitamin B_3_, 7.6 nmol/L for vitamin B_6_, 0.36 nmol/L for vitamin B_12_ (Table 1).

**Table 1:** Linearity and sensitivity of magnetic SPE.

Compound	Regression	Equation	Weighting factor	R^2^	Linearity range	LOD	LOQ	Unit
Hcy	Linear	y = 0.152x + 0.19	1/x^2^	0.9995	2.2-120	0.25	0.5	μmol/L
Met	Linear	y = 0.634x + 0.0449	1/x^2^	0.9998	3.3-180	0.2	0.7	μmol/L
folate	Linear	y = 0.142x + 0.0434	1/x^2^	0.9974	0.8-42.9	0.17	0.34	nmol/L
5-MTHF	Linear	y = 0.326x - 0.0546	1/x^2^	0.9989	1.52-82.12	0.043	0.1	nmol/L
5-FTHF	Linear	y = 1.19x + 0.129	1/x^2^	0.9992	0.74-39.85	0.042	0.25	nmol/L
vitamin B_2_	Linear	y = 0.0593x + 0.017	1/x^2^	0.9993	3.2-171.8	0.08	0.5	nmol/L
vitamin B_3_	Linear	y = 0.0546x+0.0594	1/x^2^	0.997	16.2-1317	12.2	36.5	nmol/L
vitamin B_6_	Linear	y = 0.505x + 0.0841	1/x^2^	0.9977	3.8-205.4	1.9	7.6	nmol/L
vitamin B_12_	Linear	y = 0.0439x + 0.00175	1/x^2^	0.9966	0.22-11.9	0.074	0.36	nmol/L

The accuracy was evaluated by recovery, spiking low, medium and high concentrations of standards in serum. The recoveries of Hcy, Met, folate, 5-MTHF, 5-FTHF, vitamin B_2_, vitamin B_3_, vitamin B_6_, and vitamin B_12_ were in the ranged of 86.97%-94.48%, 98.96%-104.49%, 112.01%-114.48%, 85.34%-92.43%, 85.46%-101.37%, 85.29%-92.30%, 86.76%-93.19%, 100.68%-110.70%, 85.84%-100.58%, respectively (Table 2).

**Table 2:** Recovery of magnetic SPE at low, medium and high concentrations.

	Low level(n = 5)			Medium level(n = 5)			High level(n = 5)		
	Theoretical Concentration	Recovery (%)	CV (%)	Theoretical Concentration	Recovery (%)	CV (%)	Theoretical Concentration	Recovery (%)	CV (%)
Hcy	4	89.34	2.72	24	94.48	3.88	80	86.97	5.39
Met	6	98.96	4.79	36	104.49	5.34	120	99.54	7.84
folate	1.4	112.01	2.11	8.5	114.26	0.51	28.5	114.48	0.68
5-MTHF	2.7	92.43	5.58	16.4	89.14	3.13	54.7	85.34	3.48
5-FTHF	1.3	85.46	6.57	8	95.41	2.24	26.5	101.37	1.36
vitamin B_2_	8.4	85.83	5.51	34.4	85.29	2.53	114.5	92.30	2.40
vitamin B_3_	44	86.76	5.28	263	90.01	2.85	878	93.19	3.86
vitamin B_6_	6.8	100.68	3.50	41	110.70	7.98	137	104.00	3.76
vitamin B_12_	0.4	85.84	4.03	2.4	100.58	3.45	8	91.84	8.57

Hcy and Met are in units of μmol/L, respectively. Units for other analytes are nmol/L.

The intra-assay coefficients of variation (CV) of the magnetic SPE method were 3.95%–6.81%, 2.04%–6.78%, 2.05%–7.65%, 2.50%–4.13%, 0.82%–3.12%, 1.91%–3.86%, 6.33%–7.45%, 3.30%–4.52%, 2.64%–5.16% for Hcy, Met, folate, 5-MTHF, 5-FTHF, vitamin B_2_, vitamin B_3_, vitamin B_6_, and vitamin B_12_, respectively. The inter-assay CVs of the magnetic SPE method were 3.71%–11.43%, 3.88%–8.63%, 2.95%–7.88%, 3.19%–4.11%, 1.17%–3.76%, 1.62%–4.52%, 5.05%–8.72%, 4.56%–8.93%, 3.99%–6.93% for Hcy, Met, folate, 5-MTHF, 5-FTHF, vitamin B_2_, vitamin B_3_, vitamin B_6_, and vitamin B_12_, respectively (Table 3).

**Table 3:** Precision of magnetic SPE at low, medium and high concentrations.

	Low level			Medium level			High level		
	Theoretical Concentration	intra-assay CV(%) (n = 6)	inter-assay CV(%) (n = 18)	Theoretical Concentration	intra-assay CV(%) (n = 6)	inter-assay CV(%) (n = 18)	Theoretical Concentration	intra-assay CV(%) (n = 6)	inter-assay CV(%) (n = 18)
Hcy	4	5.18	11.43	24	6.81	7.77	80	3.95	3.71
Met	6	2.04	3.88	36	6.78	8.43	120	4.46	8.63
folate	1.4	7.65	7.88	8.5	2.05	3.30	28.5	2.74	2.95
5-MTHF	2.7	2.50	4.11	16.4	4.13	3.61	54.7	2.98	3.19
5-FTHF	1.3	3.08	3.76	8	3.12	3.02	26.5	0.82	1.17
vitamin B_2_	8.4	3.86	4.52	34.4	1.91	2.58	114.5	2.10	1.62
vitamin B_3_	44	6.33	8.72	263	6.86	6.11	878	7.45	5.05
vitamin B_6_	6.8	3.74	4.56	41	4.52	6.18	137	3.30	8.93
vitamin B_12_	0.4	5.16	6.93	2.4	3.42	4.48	8	2.64	3.99

Hcy and Met are in units of μmol/L, respectively. Units for other analytes are nmol/L.

Traditional sample preparation methods for tandem mass spectrometry (such as protein precipitation, liquid-liquid extraction, and solid-phase extraction) utilize isotopic internal standards to mitigate matrix effects. This study assessed the matrix effects of the method using internal standard-normalized matrix factors, calculated as the matrix factor of the analyte divided by the matrix factor of the internal standard (matrix factor = signal intensity in 5% BSA solution/signal intensity in 50% methanol solution). The internal standard-normalized matrix factors for the analytes at low, medium, and high concentrations in both 5% BSA solutions and 50% methanol solution were all between 0.8 and 1.2. The results demonstrate that this sample preparation method effectively eliminates matrix effects in LC-MS/MS detection (Table 4). Since there is no corresponding internal standard for vitamin B_12_, the internal standard normalization matrix factor cannot be calculated.

**Table 4:** Matrix effects of magnetic SPE at low, medium and high concentrations.

	Low(n = 6)			Medium(n = 6)			High(n = 6)		
	Theoretical Concentration	IS-Normalized MF	Bias (%)	Theoretical Concentration	IS-Normalized MF	Bias (%)	Theoretical Concentration	IS-Normalized MF	Bias (%)
Hcy	4	118.18	18.18	24	105.30	5.30	80	103.78	3.78
Met	6	95.37	−4.63	36	106.08	6.08	120	112.81	12.81
folate	1.4	119.03	19.03	8.5	100.29	0.29	28.5	99.52	−0.48
5-MTHF	2.7	93.79	−6.21	16.4	85.65	−14.35	54.7	84.13	−15.87
5-FTHF	1.3	104.38	4.38	8	96.45	−3.55	26.5	96.91	−3.09
vitamin B_2_	8.4	101.13	1.13	34.4	91.15	−8.85	114.5	89.53	−10.47
vitamin B_3_	44	101.83	1.83	263	83.11	−16.89	878	82.69	−17.31
vitamin B_6_	6.8	103.16	3.16	41	97.32	−2.68	137	96.76	−3.24

Hcy and Met are in units of μmol/L, respectively. Units for other analytes are nmol/L.

To evaluate carryover, low and high concentration samples were injected alternately. The injection sequence (L1, H, L2, L3) was cycled six times, where L1, L2, and L3 were the same low concentration and H was the high concentration. Carryover was calculated as (L2-L1)/L1, and the coefficient of variation (CV) of the low concentration was determined. The carryover values were −2.86%, −7.43%, −7.83%, −1.04%, −1.77%, −0.87%, 1.23%, 0.02% and 5.21% for Hcy, folate, Met, 5-MTHF, 5-FTHF, vitamin B_2_, vitamin B_3_, vitamin B_6_, and vitamin B_12_, respectively, suggesting that carryover could be neglected. The CV of analytes were all less than 10% (Table 5).

**Table 5:** Carryover of magnetic SPE at low, medium and high concentrations.

		Hcy	Met	folate	5-MTHF	5-FTHF	vitamin B_2_	vitamin B_3_	vitamin B_6_	vitamin B_12_
Theoretical Concentration	low	2.2	3.3	0.8	1.5	0.74	3.2	24.4	3.8	0.22
	high	60	90	21.4	41	20	86	658.5	102.7	6
Carryover (%)		−2.86	−7.43	−7.83	−1.04	−1.77	−0.87	1.23	0.02	5.21
CV (%)		7.16	9.89	7.23	3.03	4.84	5.68	5.84	7.64	8.83

Hcy and Met are in units of μmol/L, respectively. Units for other analytes are nmol/L.

### 3.4 Comparative study

Using the MSPE method developed in this study and the protein precipitation method, the levels of Hcy and its eight related metabolites were measured in the same batch of serum samples. The correlation coefficients (R^2^) for all analytes were above 0.94, indicating excellent agreement between the two methods (Table 6).

**Table 6:** Correlation coefficients and regression slopes of analytes.

analytes	correlation coefficients	regression slopes
Hcy	0.9632	0.9634
Met	0.9789	0.9580
folate	0.9886	0.9259
5-MTHF	0.9838	0.9843
5-FTHF	0.9462	0.8449
vitamin B_2_	0.9916	0.9737
vitamin B_3_	0.9957	0.9766
vitamin B_6_	0.9829	0.9537
vitamin B_12_	0.9930	0.9745

**Fig. 7:**
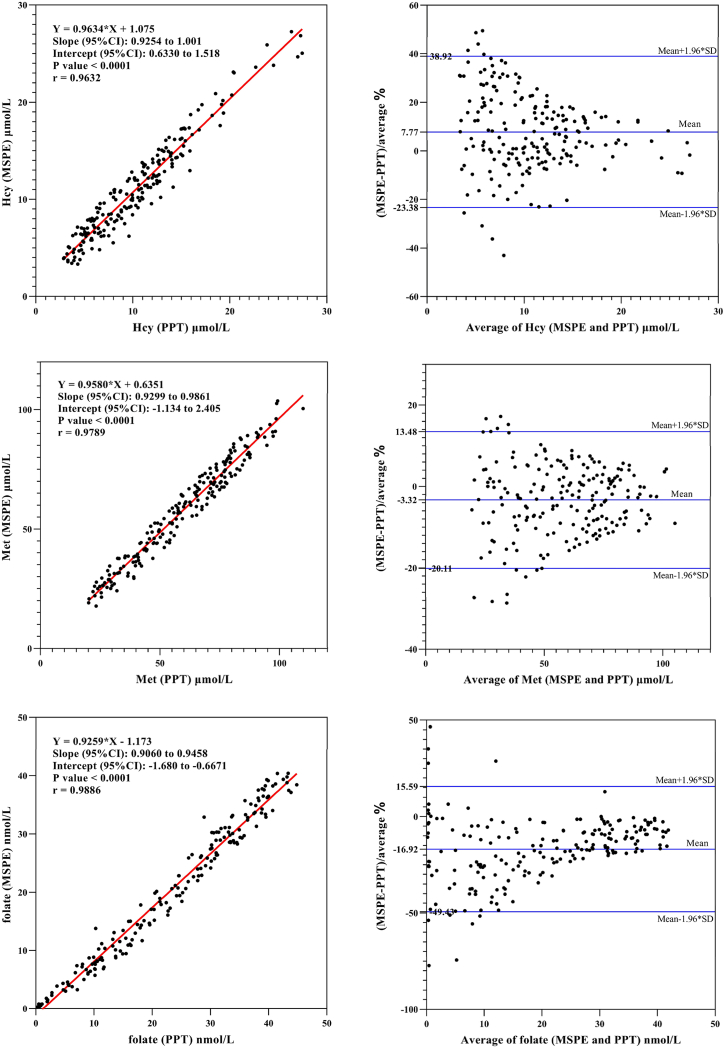

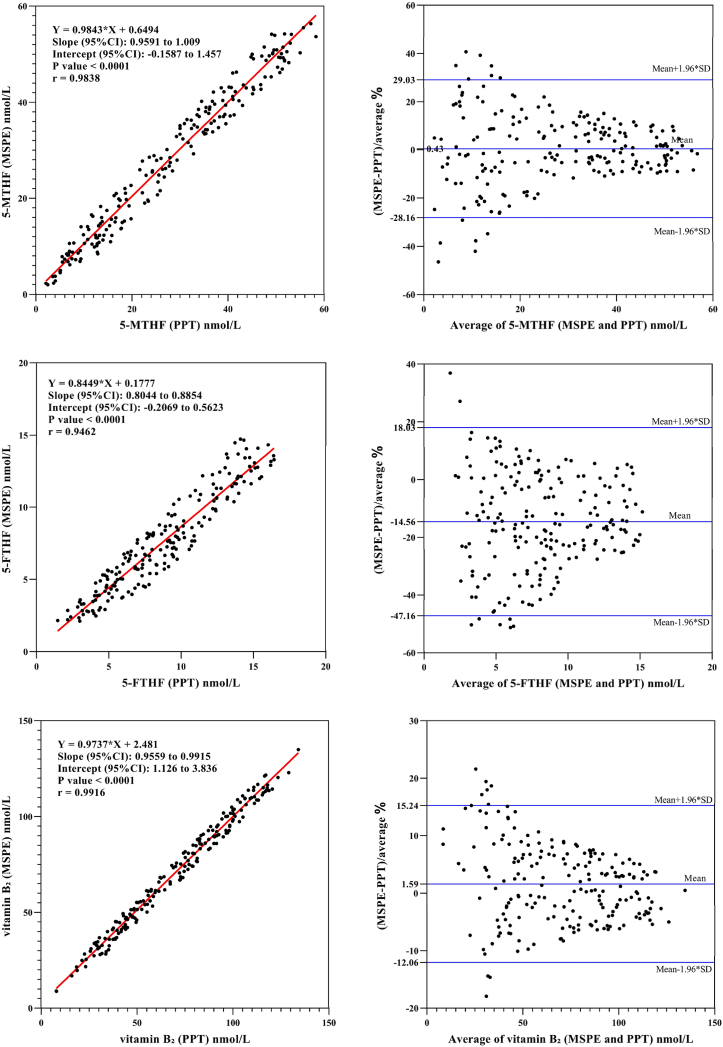

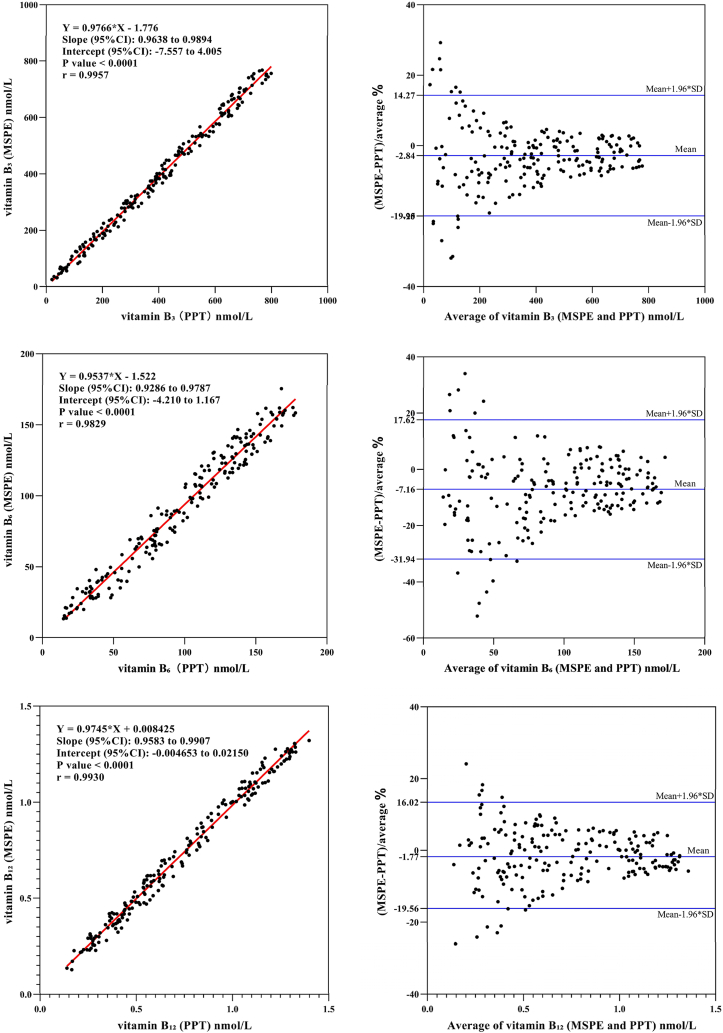
Comparison of the results between magnetic solid-phase extraction (MSPE) and protein precipitation (PPT) for Hcy, methionine, folate, 5-MTHF, 5-FTHF, vitamin B_2_, vitamin B_3_, vitamin B_6_, and vitamin B_12_.

Passing-Bablok regression analysis indicated no systematic bias between the two methods. Compared with the traditional manual protein precipitation method, the MSPE method significantly enhances the level of semi-automatization and throughput in sample pretreatment while maintaining excellent analytical performance (Fig. 7).

The present study developed an automated sample preparation method for LC-MS/MS that enables simultaneous quantification of nine Hcy-related metabolites in serum. This method is specific, sensitive, achieves automated sample processing, and effectively overcomes matrix effects, providing an efficient analytical solution for clinical precision medicine.

In mass spectrometry-based detection methods, serum contains abundant proteins, phospholipids, salts, and endogenous metabolites, which can cause interference such as ion suppression or ion enhancement during sample analysis [23], thereby affecting the accuracy, precision, and sensitivity of quantification [24]. The systemic monitoring of Hcy, folate, 5-MTHF, 5-FTHF, methionine, vitamin B_2_, vitamin B_3_, vitamin B_6_, and vitamin B_12_ is of significant importance for the management of related diseases [25], including cardiovascular diseases, neural tube defects, cognitive impairment/Alzheimer's disease, homocystinuria, megaloblastic anemia, and peripheral neuropathy. However, the concentration ranges of these analytes span widely from μmol/L to nmol/L levels, imposing stringent requirements on the sample pretreatment method in terms of broad dynamic range purification and enrichment capability [26]. The magnetic solid-phase extraction (MSPE) method employed in this study utilizes HLB magnetic beads to selectively bind with target analytes, enabling efficient removal of phospholipids, proteins, and other interfering substances, and effectively overcoming matrix effects [27]. Through optimized conditions, this method achieves effective enrichment even for very low concentrations of vitamin B_12_, meeting the requirements for simultaneous detection of analytes across a wide concentration range (from μmol/L to nmol/L). Additionally, magnetic beads can be functionalized with various modifications to bind and enrich other target analytes [28], highlighting the high flexibility and expandability of the magnetic solid-phase extraction approach.

The non-specific HLB magnetic beads used in this study for enriching the target analytes do not require coupling with other immunosorbents [28](such as antibodies [29], molecularly imprinted polymers, or oligonucleotide adsorbents). HLB magnetic beads are low-cost and readily available. With only 5 mg of HLB magnetic beads used per sample for analyte enrichment, the overall cost of the method is relatively low, which facilitates its clinical implementation and application [30]. Compared to the protein precipitation method [31], the sensitivity of magnetic solid-phase extraction is slightly lower than that of protein precipitation but higher than that of immunoassays. Moreover, the limits of detection and quantification are fully adequate for analyzing both healthy individuals and patients. Simultaneously, the magnetic bead-based pretreatment offers cleaner sample processing (with fewer interferences and lower background noise) [30]. Furthermore, it can be integrated with pre-packaging technology and automated liquid handling systems to achieve full automation [32].

Hyperhomocysteinemia is an independent risk factor for stroke and plays a crucial role in the diagnosis, treatment, and prevention of this condition [33]. Meanwhile, homocysteine and its nine related metabolites are associated with various diseases including cardiovascular diseases, neurological disorders, reproductive system diseases, tumors, diabetes, and chronic kidney disease [34]. Moreover, the combined detection of these biomarkers holds greater clinical value than single-marker testing [35]. In contrast, immunoassays are limited to quantifying only one biomarker per analysis and exhibit relatively low sensitivity [17], while the protein precipitation method suffers from low automation [20]. Therefore, with its semi-automatization and high-throughput characteristics, the present method enables systematic monitoring of the "metabolic status" of homocysteine, providing comprehensive and accurate support for the auxiliary diagnosis, treatment, and prevention of related diseases.

In summary, the magnetic solid-phase extraction pretreatment method established in this study integrates matrix cleanup, high throughput, semi-automatization, high sensitivity, and a wide detection range. This approach offers a robust and reliable solution for the simultaneous analysis of multiple classes of biomarkers.

## 4 Conclusion

This study developed an automated MSPE method of LC-MS/MS capable of simultaneously quantifying serum levels of Hcy, folate, 5-MTHF, 5-FTHF, methionine, vitamin B_2_, vitamin B_3_, vitamin B_6_, and vitamin B_12_. With advantages in semi-automatization and high throughput, it provides an efficient analytical tool for clinical precision medicine.

## CRediT authorship contribution statement

**Zhicheng Ye:** Writing – original draft, Visualization, Validation, Methodology, Investigation, Conceptualization. **Xueding Han:** Validation, Methodology. **Guanfeng Lin:** Supervision, Conceptualization. **Jianwei Zhou:** Writing – review & editing, Supervision, Resources, Conceptualization. **Yingsong Wu:** Writing – review & editing, Validation, Supervision, Project administration, Funding acquisition, Formal analysis.

## Disclosure

The authors have nothing to disclose.

## Declaration of competing interest

These authors declare that they have no known competing financial interests or personal relationships that could have appeared to influence the work reported in this paper.

## Data Availability

Data will be made available on request.
