# Supplementary material for: A semi-automatization magnetic solid-phase extraction method of LC-MS/MS for the quantification of homocysteine and its related metabolites in serum
Source: Pract Lab Med. 2026 May 5;50:e00535. doi: 10.1016/j.plabm.2026.e00535 (PMC13224127; doi:10.1016/j.plabm.2026.e00535)
Supplement: Multimedia component 1 [file mmc1.docx]

**Supporting Information**

**A semi-automatization magnetic solid-phase extraction method of LC-MS/MS for the quantification of** **homocysteine and its related metabolites in serum**

Zhicheng Ye^1^, Xueding Han^1^, Guanfeng Lin^1^, Jianwei Zhou^2^*, and Yingsong Wu^1^*

^1^Institute of Antibody Engineering, School of Laboratory Medicine and Biotechnology, Southern Medical University, Guangzhou 510515, China

^2^Guangzhou Darui Biotechnology Co., Ltd., Guangzhou 510705, China

*** Corresponding authors:**

**Yingsong Wu** − Institute of Antibody Engineering, School of Laboratory Medicine and Biotechnology, Southern Medical University, Guangzhou 510515, China; orcid.org/0000 0002-3734-702X; Phone: +86-20-62789355; Email: wg@ smu.edu.cn; Fax: +86-20-37247604

**Jianwei Zhou** − Guangzhou Darui Biotechnology Co., Ltd., Guangzhou 510705, China; Phone: +86-20-32299700; Email: 396763629@qq.com

**Table of Contents**

**Section Ⅰ. Supporting Figures**

**Figure S1.** Automatic nucleic acid extractor Stream SP96

**Section Ⅱ. Supporting Tables**

**Table S1.** Automatic sample preparation procedure for the magnetic bead extraction method

**Table S2.** Gradient elution procedure

**Table S3.** MRM parameters for measuring homocysteine and its related metabolites

**Figure S1.** Automatic nucleic acid extractor Stream SP96
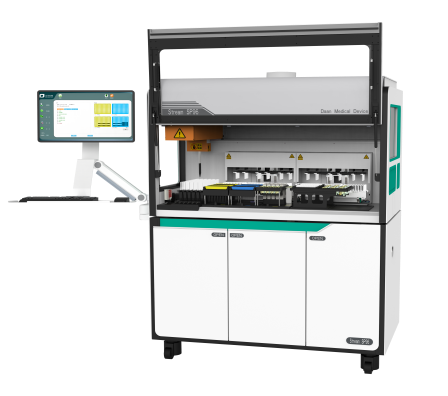


**Table S1.** Automatic sample preparation procedure for the magnetic bead extraction method

| Procedure | Well position | Operation | Mixing time (min) | Mixing speed | Solution volume (µl) |
| --- | --- | --- | --- | --- | --- |
| 1 | Line 1 | Activating | 5 | Middle | 300 |
| 2 | Line 2 | Equilibrating | 5 | Middle | 300 |
| 3 | Line 3 | Enriching | 10 | Middle | 650 |
| 4 | Line 4 | Washing 1 | 5 | Middle | 300 |
| 5 | Line 5 | Washing 2 | 5 | Middle | 300 |
| 6 | Line 6 | Eluting | 10 | Middle | 100 |

**Table S2.** Gradient elution procedure

| time | flow | A% | B% |
| --- | --- | --- | --- |
| 0 | 0.8 | 99 | 1 |
| 3 | 0.8 | 50 | 50 |
| 3.2 | 0.8 | 2 | 98 |
| 4.2 | 0.8 | 2 | 98 |
| 4.5 | 0.8 | 99 | 1 |
| 6 | 0.8 | 99 | 1 |

**Table S3.** MRM parameters for measuring homocysteine and its related metabolites

| No. | Compound | Parent (m/z) | Daughter (m/z) | DP | EP | CE | CXP |
| --- | --- | --- | --- | --- | --- | --- | --- |
| 1 | VB3 | 123 | 80.1 | 65 | 65 | 27 | 8 |
| 2 | VB3-IS | 127.1 | 83.1 | 65 | 65 | 27 | 8 |
| 3 | VB6 | 184 | 148.1 | 40 | 40 | 27 | 8 |
| 4 | VB6-IS | 187 | 150.1 | 40 | 40 | 27 | 8 |
| 5 | 5-MeTHF | 460.3 | 313.3 | 40 | 40 | 27 | 6 |
| 6 | 5-MeTHF-IS | 464 | 317.3 | 48 | 48 | 27 | 6 |
| 7 | VB2 | 377.2 | 243.3 | 90 | 90 | 40 | 2.1 |
| 8 | VB2-IS | 383.2 | 249.3 | 90 | 90 | 38 | 7 |
| 9 | Met | 150.1 | 104.1 | 40 | 40 | 14 | 3 |
| 10 | Met-IS | 153.1 | 107.1 | 40 | 40 | 14 | 3 |
| 11 | HCY | 136.1 | 90.1 | 40 | 40 | 15 | 8 |
| 12 | HCY-IS | 140.1 | 94.1 | 40 | 40 | 15 | 8 |
| 13 | FA | 442.2 | 295.2 | 40 | 40 | 21 | 9 |
| 14 | FA-IS | 447.2 | 295.2 | 40 | 40 | 21 | 9 |
| 15 | 5-FoTHF | 474.3 | 327.2 | 40 | 40 | 28 | 9 |
| 16 | 5-FoTHF-IS | 478.3 | 331.2 | 40 | 40 | 28 | 9 |
| 17 | B12 | 678.2 | 147.1 | 100 | 7 | 39 | 11 |
